# How the COVID-19 Pandemic Changes the Subjective Perception of Meaning Related to Different Areas of Life in Austrian Psychotherapists and Patients

**DOI:** 10.3390/ijerph17228600

**Published:** 2020-11-19

**Authors:** Elke Humer, Wolfgang Schimböck, Ida-Maria Kisler, Petra Schadenhofer, Christoph Pieh, Thomas Probst

**Affiliations:** 1 Department for Psychotherapy and Biopsychosocial Health, Danube University Krems, 3500 Krems, Austria; elke.humer@donau-uni.ac.at (E.H.); christoph.pieh@donau-uni.ac.at (C.P.); 2 ABILE-Viktor Frankl Education Austria, 3390 Melk, Austria; wolfgang.schimboeck@liwest.at (W.S.); dr.i.kisler@gmail.com (I.-M.K.); petra.schadenhofer@gmail.com (P.S.); 3 Telephone Emergency Service—Lower Austria (TelefonSeelsorge NÖ), Diocese St. Pölten, 3100 St. Pölten, Austria

**Keywords:** meaning, COVID-19, psychotherapy, work, health

## Abstract

We assessed psychotherapists’ and patients’ ratings of their subjective perception of meaning related to different areas of life before the COVID-19 pandemic as compared to the time during the COVID-19 pandemic. In a quantitative cross-sectional study, Austrian psychotherapists (*N* = 222) were recruited by e-mail, who in turn recruited their patients (*N* = 139). Therapists and patients were asked to rate the meaning of different areas of life before as well as during the COVID-19 crisis. The psychotherapists showed an overall higher rating of the importance of areas of life compared to their patients (*p* < 0.001). The rating of the importance of the domains of living was differently affected by the COVID-19 situation (*p* < 0.001). While the meaning of physical and mental health during COVID-19 was rated higher than before, the opposite was observed for work (*p* < 0.001). No differences were found for relationships and friends, as well as for hobbies. As no interactions between perspective (therapists vs. patients), area of life, and time point (before vs. during COVID-19) were observed, it can be concluded that the COVID-19 situation changed the subjective attribution of meaning concerning different aspects of life similarly in therapists as well as patients. While mental and physical health gained subjective importance, the opposite was observed for work.

## 1. Introduction

The novel coronavirus disease (COVID-19), a novel disease resulting in severe acute respiratory syndrome caused by coronavirus 2 (SARS-Cov-2), confronts humanity with an unquestionable, unprecedented crisis [1]. The COVID-19 pandemic and the resulting measures to mitigate the virus circulation dramatically affect health, economics, and social connections around the world [2]. This public health challenge has provoked inconvenience, anxiety, and uncertainty and awakened humans to their existence [3,4]. Viktor Frankl, Austrian neurologist and psychotherapist, founder of logotherapy and existential analysis in the 1950s, would call this current state human beings are in a veritable “existential frustration” [5] which may lead some of us to noogenic neuroses. Frustration of meaning in life, according to logotherapy, may appear in the impossibility to fulfill work (loss of work during these days of the pandemic) or a deed, impossibility to live encountering (social activities, free traveling, etc.), and last but not least, loss of basic trust producing fear of life [5].

The COVID-19 crisis has not only explicitly questioned the capacity to survive—the most fundamental human need [6]—but also questioned the illusion of job security [7]. Furthermore, the strong restrictive measures against the rapid spread of the virus—as decided by most countries around the world—have led to substantial adverse effects on the global economy, causing a strong worldwide increase in the unemployment rate [8]. Employment status is not only necessary to provide the means for survival, such as ensuring access to food, shelter, housing, clothing, and safety, but can also fulfill other needs, such as perception of self-esteem and self-efficacy. According to the psychology of working theory [9], working can also provide access to the social world and fulfill the need for social connection and contribution, and self-determination. Therefore, the loss of work represents a source of existential fear [7]. The fundamental importance of work for human beings is further supported by the finding that among several types of disasters (e.g., natural disasters, war, epidemics/pandemics, economic recession), economic recessions most significantly impact mental well-being, even going along with increased suicide rates [10]. In this regard, recent predictions estimate that the increase in unemployment rates related to the COVID-19 crisis will strongly enhance suicides up to about 10,000 per year [11]. While the pandemic is associated with job loss in several professions, psychotherapists might even face a higher workload in the long term due to the increase in mental symptoms in the general population [12,13]. In the short term, the need to adapt the format of how psychotherapy is provided might pose a specific challenge (i.e., to provide psychotherapy via the internet or telephone with all its technical and legal challenges [14,15,16]). We recently reported that during the first weeks of the COVID-19 lockdown in Austria, the average number of patients treated per week decreased and face-to-face sessions were largely substituted by sessions via telephone or internet [15]; however, despite these challenges psychotherapists were exposed to in the early weeks of the COVID-19 outbreak in Austria, job-related worries and fears of existence, as well as stress level, were neither affected by the extent of the reduction in the number of patients treated nor by the format of how psychotherapy was provided during the COVID-19 lockdown in Austria [17].

Besides detrimental effects on economics, several previous studies highlight that many psychological problems emerged progressively during this state of public health emergency [18]. A high prevalence of mental symptoms during the COVID-19 pandemic, including in those at the forefront of healthcare [19], has been observed in many studies [20]. For example, a meta-analysis based on 9074 participants found a prevalence of 32% for anxiety symptoms and 34% for depression [12]. Consequently, an increase in mental health care utilization can be expected in the near future. Suggested causes for high rates of mental health issues during COVID-19 are job insecurity and the fear of infection [21]. Also, physical distancing and isolation due to governmental restrictions to prevent uncontrolled spreading can impact mental health [20]. Forced isolation, reduction of social contacts, and risk of domestic violence range among the most important risk factors for psychological distress individuals are exposed to during the COVID-19 pandemic [22].

In addition to consequences on mental health, the COVID-19 pandemic has been reported to be related to detrimental behavioral alterations and negative impacts on physiological health. In this regard, dramatic shifts in substance use (increased alcohol consumption and smoking), physical activity (physical inactivity and higher rates of sedentary behavior), and diet (poor diet and too high energy intake) have been reported, which likely also interact with mental health [23].

Social behavior is also dramatically affected by the COVID-19 pandemic, as the reduction of social contacts is considered as one of the most important measures to fight the spread of the virus. In general, social behavior represents an important protective factor and has been associated with mental health, such as depression, stress, and anxiety [24], as well as physical health (i.e., physical activity, alcohol consumption, smoking, body mass index) [25]. Therefore, social relationships are important for mental and physical functioning [26]. Also, relationship quality is related to mental health during COVID-19 with the best mental health shown by those having a good relationship quality [27]. Social distancing measures on the one hand force people to live closer together with some people, but further apart from others. In particular, the lockdown measures have necessitated close, constant contact with partners and families, but also isolated individuals from their friends, acquaintances, and wider communities.

To summarize, the COVID-19 pandemic poses far-reaching challenges in almost all aspects of life. Following existential-humanistic psychology’s focus on dialectics and paradox [28], the Chinese idiom reminds us that “crisis” does not only refer to danger but also an opportunity [1,3]. A large-scale crisis, such as a pandemic, takes mankind out of the routines and brings individuals to reflect on what they take for granted. Such catastrophes typically also call habitual patterns of thinking, experiencing, relating, and behaving into question [1]. Therefore, this crisis not only poses challenges but also offers opportunities for personal and collective growth as highlighted recently [1]. As an example, the increased time spent at home could present individuals with newly discovered opportunities to discover interests [29]. The purpose literature highlights that challenging experiences can be transformed into opportunities [30,31]. Thus, people might also be able to transform this global adversity into purpose, in considered values and perceived meaningfulness [29]. As each situation in life represents a challenge to individuals and represents a problem for them to solve, the question of the meaning of life may be reversed. People should not ask what the meaning of their life is, but rather they must recognize that it is they who are asked. To life, they can only respond by being responsible. People are capable of changing the world for the better if possible, and of changing themselves for the better if necessary [5]. In general, a committed sense of purpose is regarded as an important fuel to preserve when faced with hardship [30,31], which might also make people feel the countless challenges posed by COVID-19 more tolerable and meaningful [29].

However, whether the COVID-19 crisis is associated with changes in the perception of the meaning of life (i.e., how important individuals regard certain areas of life) has not been investigated so far. Furthermore, whether potential changes in the perception of the importance of different areas of life due to COVID-19 differ among individuals with mental disorders (patients) as compared to those who are assumed to be well aware of a positive, mentally healthy lifestyle (psychotherapists) has not been investigated yet.

This paper aims to examine the changes in the rating of the importance of certain areas of life in Austrian psychotherapists and patients during the COVID-19 crisis. In Austria, the first COVID-19 cases were reported on the 25 February 2020 and a nationwide curfew went into force from the 16 March 2020 until the 30 April 2020. After a peak of the daily confirmed COVID-19 cases at the end of March 2020 (>1000 confirmed cases per day), with the end of the lockdown, daily cases decreased and remained at a low level (<100 cases/day) until the end of June 2020. From July to August 2020, daily cases started to increase again [32]. The survey on which the current study is based was started on the 26 June 2020 and was open until the 3 September 2020.

The following research questions (RQs) were investigated:

RQ1: Is there a difference in the subjective perception of the meaning of certain areas of life during the COVID-19 crisis as compared to the time before COVID-19 in psychotherapists and their patients?

RQ2: Is there an age difference in the rating of the subjective importance of different areas of life before and during COVID-19?

RQ3: Is there a gender difference in the rating of the subjective importance of different areas of life before and during COVID-19?

## 2. Materials and Methods

### 2.1. Study Design

To investigate our research questions, a quantitative cross-sectional study was conducted in the form of an online survey using the platform REDCap. The information about the survey including the link was sent by the Austrian Federal Association for Psychotherapy (ÖBVP) to their members at the end of June 2020. Additionally, e-mail addresses were exported from the official list of psychotherapists (around 9000 registered psychotherapists in July 2020) to contact them.

The present study was part of a larger survey in which psychotherapists were asked to participate and also to invite their patients to participate if both experienced a switch in the psychotherapy format (either from remote psychotherapy to face-to-face psychotherapy or the other way around). Both surveys (therapists’ and patients’ versions) were open from the 26 June 2020 until the 3 September 2020. In total, 222 psychotherapists and 139 patients completed the survey. All the participating therapists and patients were included in further analyses, irrespective of whether they stated in the survey changes of the treatment format during psychotherapy.

Therapists were offered continuing education credit points to compensate for the time spent conducting the survey and to motivate them to participate. Patients’ participation was voluntary, without incentives. Participants had to agree to the data protection declaration to start the survey (electronic informed consent). The principles outlined in the Declaration of Helsinki were followed and the ethics committee of the Danube University Krems (Austria) approved the study.

### 2.2. Measures

Psychotherapists reported their age, gender, and level of qualification (registered in the official list of licensed therapists vs. psychotherapists in training under supervision), as well as their therapeutic orientation.

Patients were asked about their age, gender, and months receiving psychotherapy. No information was obtained regarding the therapeutic orientation of the psychotherapy the patients received, as patients were not matched to the therapists in the online survey to ensure anonymous data collection.

To examine the patients’ mental health problems, the ICD-10 Symptom Rating (ISR) [33] was administered to the patients. The ISR comprises 29 items, which are rated on a five-point Likert scale. The ISR enables the calculation of a global score and five syndrome scores (i.e., depression (four items), anxiety (four items), obsessive-compulsive (three items), somatoform (three items), eating (three items)).

Patients, as well as therapists, were asked to rate their subjective perception of the meaning of six domains of living/values on a five-point scale from “not important at all” (coded as 1) to “extremely important” (coded as 5): (1) work, (2) relationships, (3) acquaintances, friends, (4) leisure time, hobbies, (5) physical health, (6) mental health.

First, participants were asked to rank the importance of those areas of life before the current corona crisis and thereafter they had to rank them during the current COVID-19 crisis.

### 2.3. Statistics

For statistical analysis, the IBM SPSS (IBM Corporation, Armonk, NY, USA) Statistics 26 software program was used.

To analyze possible differences in sociodemographic characteristics between therapists and patients, chi-squared tests and *t*-tests were applied.

Statistics for RQ1: Mixed ANOVAs were performed to investigate whether the subjective perception of meaning changed during COVID-19 vs. before COVID-19, whether the rating of the meaning differed among areas of life and between therapists and patients. Moreover, we investigated possible two- and three-way interactions between time point, areas of life, and perspective (patients vs. therapists). In this ANOVA, the subjective rating of the meaning was the dependent variable. There were two within-subject factors: the first was “change” (two levels: during COVID-19, before COVID-19) and the second was “area” (six levels: (1) work, (2) relationships, (3) acquaintances, friends, (4) leisure time, hobbies, (5) physical health, (6) mental health). There was one between-subject factor, that is, “perspective” (two levels: therapist, patient). All main effects (ME) and interaction effects (IE) were examined. The Greenhouse–Geisser corrected values are presented. Bonferroni-corrected simple effects tests were conducted for significant ME and IE.

Statistics for RQ2: To answer the research questions of whether age affects the subjective perception of the meaning of different areas of life before or during COVID-19 or the change in the subjective meaning during vs. before COVID-19, we calculated Pearson correlations.

Statistics for RQ3: To evaluate potential gender differences, only female and male participants were included, as only one transgender patient participated in the study. Differences between female and male participants were calculated by *t*-tests comparisons. Bonferroni correction for multiple comparisons was applied for results interpretation of RQ3, considering *p* < 0.0028 as significant (*p* < 0.05/18 *t*-tests).

All statistical tests were performed two-tailed and the significance level was set to *p* < 0.05 before Bonferroni correction.

## 3. Results

### 3.1. Participants

In total, 139 Austrian patients and 222 Austrian therapists participated in the online survey. There were no differences concerning gender between therapists and patients (Table 1). However, therapists were on average 11.38 years older than their patients (*p* < 0.001). Therapists already registered in the list of psychotherapists (*n* = 202) were on average 11.69 (SD = 9.41) years in the profession. The remaining 20 therapists were advanced psychotherapy students already allowed to practice psychotherapy under supervision.

The distribution of their psychotherapeutic orientations was as follows: psychodynamic 22.1% (*n* = 49), humanistic 46.4% (*n* = 103), systemic 20.7% (*n* = 46), behavioral 10.8% (*n* = 24).

At the time of their participation in the survey, patients already received psychotherapeutic treatment for on average 21.42 (SD = 18.76) months (range from 0 to 150 months). Analysis of the ISR revealed a global score of M = 1.14 (SD = 0.69), indicating moderate symptom distress [33]. The five ISR syndrome scores indicate low syndrome distress on each scale and were as follows: depression: M = 1.74 (SD = 0.98), anxiety: M = 1.58 (SD = 1.12), obsessive-compulsive: M = 1.28 (SD = 1.15), somatoform: M = 0.61 (SD = 0.89), eating: M = 0.85 (SD = 1.05).

### 3.2. Results for RQ1

- Differences between patients and therapists:

The rating of the meaning of life over all areas was higher in therapists (M = 4.113, SE = 0.036) as compared to their patients (M = 3.876, SE = 0.045) (ME “perspective” F(1; 359) = 16.788; *p* < 0.001).

- Differences between areas of life:

The meaning of the different areas irrespective of time point and perspective differed significantly (ME “area” F(4.135; 359) = 96.339; *p* < 0.001). Bonferroni-corrected post hoc tests revealed that the area mental health (M = 4.46, SE = 0.037) was rated highest, differing significantly from all other areas (*p* ≤ 0.002). The areas relationships (M = 4.25, SE = 0.050) and physical health (M = 4.23, SE = 0.042) followed. The area friends (M = 3.88, SE = 0.046) ranked in the fourth place, differing from all other areas (*p* < 0.001). The lowest rankings were measured for the areas work (M = 3.61, SE = 0.046) and hobbies (M = 3.53, SE = 0.050), which did not differ from each other (*p* = 1.000).

- Changes during COVID-19 as compared to before COVID-19:

The overall rating of the meaning of life did not change from before COVID-19 to during COVID-19 (ME “change” F(1; 359) = 2.216; *p* = 0.137).

**Figure 1:**
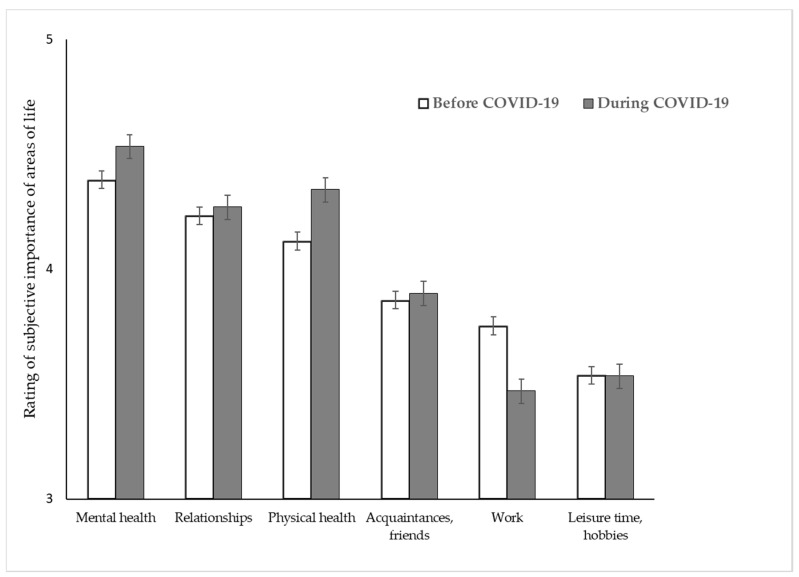
Rating of the subjective perception of the meaning of different areas of life before vs. during COVID-19 among groups (psychotherapists and patients). Note: Participants were asked to rate the subjective meaning of the different areas of life from “not important at all” (coded as 1) to “extremely important” (coded as 5).

- Changes of the ranking of areas during COVID-19 as compared to before COVID-19:

A significant interaction was found between the change and the area (IE “change × area” F(3.706; 6.945) = 22.563; *p* < 0.001) as illustrated in Figure 1.

Pairwise comparisons of each area between both time points revealed that the areas work, physical health, and mental health were rated differently before vs. during COVID-19. While the subjective rating of the meaning of work decreased during COVID-19 as compared to the times before the pandemic (*p* < 0.001), the opposite was observed for physical health (from *p* < 0.001) and mental health (*p* < 0.001).

- Differences in the change of meaning of different areas during COVID-19 as compared to before COVID-19 between patients and therapists:

The perspective interacted with neither the change (IE “change × perspective” F(1; 359) = 1.383; *p* = 0.240) nor the area (IE “area × perspective” F(4.135; 1484.367) = 1.987; *p* = 0.092).

**Figure 2:**
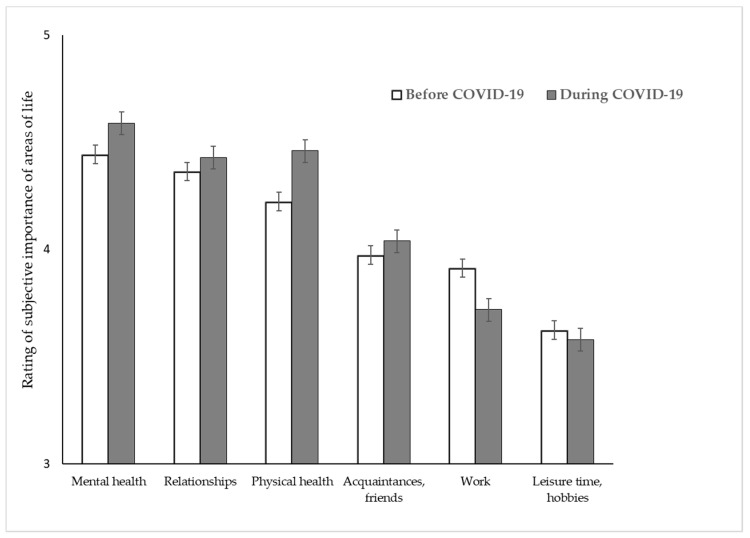
Rating of the subjective perception of the meaning of different areas of life before vs. during COVID-19 in psychotherapists. Note: Therapists were asked to rate the subjective meaning of the different areas of life from “not important at all” (coded as 1) to “extremely important” (coded as 5).

**Figure 3:**
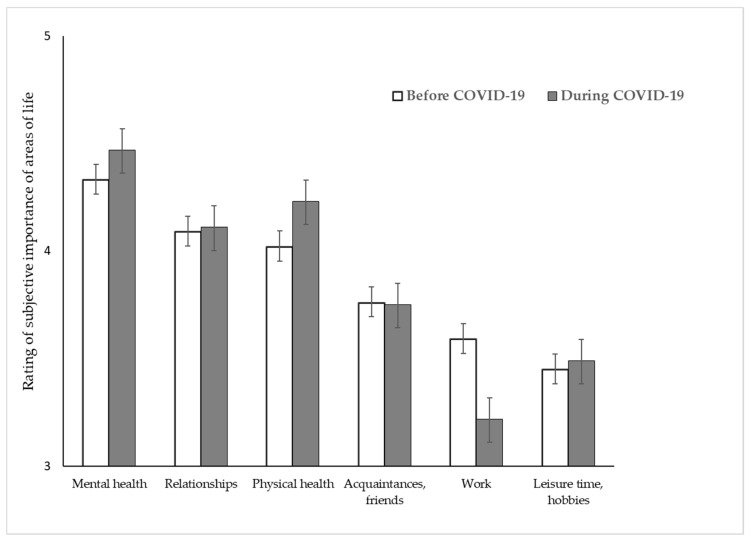
Rating of the subjective perception of the meaning of different areas of life before vs. during COVID-19 in patients. Note: Patients were asked to rate the subjective meaning of the different areas of life from “not important at all” (coded as 1) to “extremely important” (coded as 5).

No three-way interaction emerged between change, area, and perspective (IE “change × area × perspective” (F(3.706; 1330.33) = 1.332) *p* = 0.258. Descriptive statistics (mean and standard errors) for all areas and time points for psychotherapists and patients are summarized in Figure 2 and Figure 3.

### 3.3. Results for RQ2

Results of the correlation analyses to reveal potential age differences in the subjective meaning of different areas of life before COVID-19 and during COVID-19, as well as in the change from COVID-19 to the times before COVID-19, are summarized in Table 2. The change in the rating was calculated by subtracting the respective rating before the pandemic from the rating during COVID-19. Therefore, a positive value means an increase in the subjective meaning of the specific area during COVID-19 as compared to the time before the pandemic. Correlations were calculated for therapists and patients separately, as significant differences between both groups in age (Table 1) as well as in their rating of the meaning of different areas were observed.

Among therapists, a positive correlation between age and work was found before COVID-19 (r = 0.183), but not during COVID-19. This means that the older therapists were, the higher they rated the importance of work before COVID-19. The negative correlation between age and the changes in the meaning of work (*r* = −0.174) means that with the increasing age of the therapists, the subjective meaning of the importance of work decreased during COVID-19 as compared to the months before. Before the COVID-19 crisis, younger therapists regarded leisure time and hobbies as more important than older ones (*r* = −0.166), whereas no age effect for the subjective rating of the meaning of this area of life was found during COVID-19.

Among patients, younger age was associated with higher importance of leisure time and hobbies before (*r* = −0.190) as well as during (*r* = −0.168) the COVID-19 crisis. Physical health was regarded as more important by older patients before the crisis (*r* = 0.194), whereas the increase in the importance of physical health decreased with increasing age (*r* = −0.170).

### 3.4. Results for RQ3

Results for the *t*-tests comparing male and female participants concerning the meaning of different areas of life before COVID-19 and during COVID-19, as well as the change from COVID-19 to the times before COVID-19, revealed a significant difference for the area friends (t(358) = 3.214; *p* = 0.001). Women rated the importance of friends before the pandemic higher (M = 3.97, SD = 0.871) than men (M = 3.63, SD = 0.857). However, this was only significant when analyzed for the total sample (patients + therapists). For all other variables, no significant differences between male and female participants were observed when analyzed for the total sample (*p* ≥ 0.043). Separate analyses per perspective (therapists and patients) revealed no significant gender differences (*p* ≥ 0.003).

## 4. Discussion

This study evaluated whether the COVID-19 pandemic was associated with changes in the perception of the meaning related to different areas of life in psychotherapists and their patients. Additionally, potential associations with gender and age were evaluated.

We were able to show that among patients and therapists, work was regarded as less important during the COVID-19 pandemic than before. During COVID-19, unemployment rates strongly increased in Austria. Furthermore, many people had to switch to short-time working, a state-regulated system that aims to support companies to avoid laying off any of their employees instead of reducing employees’ working hours, with the government making up some of the employees’ lost income. Therefore, our findings suggest that with the increase in job insecurity and reduced time spent with professional activities, the subjective meaning of work declined. However, it has to be noted that with the current survey, we cannot rule out whether the occupational status of the participants changed due to the COVID crisis. Our findings are also not generalizable to other countries with other social support systems. In this regard, it has been observed that the economic crisis in 2008 went along with increased suicide rates, except for countries with active supportive labor-market programs such as Finland and Sweden [10,34]. Therefore, social supports and low-threshold access to emergency services (e.g., anonymous and free use of telephone crisis counseling as provided by Austrian emergency call 142) might be successful in preventing detrimental effects of the economic crisis related to the COVID-19 pandemic on mental health.

A study conducted on Hong Kongers during the SARS epidemic revealed that greater mental health awareness, positive lifestyle changes, and better social and family support were positive consequences within the broader suffering of SARS [35]. A recent longitudinal study conducted in Switzerland revealed that in students, the COVID-19 pandemic caused a shift in stressors from fears of missing out on social life to worries about their health, family, and friends [36]. However, in our study, only the subjective rating of the meaning of health changed due to the pandemic. A higher rating for physical as well as mental health was reported during the crisis compared to the time before, while no differences were found for relationships, friends, and hobbies.

A public health emergency increases the awareness of our own mortality and the mortality of our friends and family. Additionally, the pandemic is associated with the uncertainty of who will fall ill and when the crisis will end [4]. This supports the finding that physical and mental health was rated as more important during COVID-19 than before. As an infectious disease, COVID-19 might cause fear of infection and possibly severe consequences on physical health up to death [21]; therefore, results suggest that being confronted with the pandemic and its health-threatening consequences increased the subjective attribution of the meaning of physical health. Next to physical health, mental health also gained subjective importance. This is also supported by several recent studies observing an increase in mental health problems during the COVID-19 pandemic, including an increase in depressive, anxiety, and insomnia symptoms [12,13].

During these challenging circumstances, meaning might not always be readily apparent [4]. In the context of disasters, global beliefs about the world and one’s overreaching goals may be violated or even shattered [37]. Changes in the subjective meaning of life due to these challenging times do not only affect people seeking support in psychotherapy but also psychotherapists themselves, as observed in the current study. Although therapists showed a general higher rating of the meaning of certain areas of life (i.e., work, relationships, friends, hobbies and health) than their patients did, reported changes due to the COVID-19 crisis did not differ from their patients. Therefore, our results highlight that within the COVID-19 pandemic, both the therapists and the patients find themselves in considerably similar situations in terms of changes in the subjective meaning of life.

Therefore, concerning the special situation around the pandemic, the role of the therapist might become more like a guide, accompanying the patient creating meaning and making sense out of this new situation context. As not only the patient, but rather the whole of mankind is undergoing this situation for the first time, understanding of its meaning should be shared between patient and therapist in a continuous, emergent, and idiosyncratic process. Being an expert for mental disorders and allocating diagnosis seems to be less relevant in this unprecedented situation. Therefore, existential therapeutic concepts, such as logotherapy and existential analysis, seem to be of particular importance, as the current state of the pandemic demands not only a categorical diagnosis of psychopathology, but also a special focus on the humanity shared between the patient and the therapists. In this regard, existential approaches might offer a path forward into the unsure future of the life after the pandemic [4].

The following limitations have to be considered when interpreting the results: We performed a cross-sectional study, which implies that there might be a recall bias regarding the retrospective ratings of the psychotherapists and patients on the meaning of certain areas of life. A second measurement point before the COVID-19 pandemic would be necessary to investigate changes in the subjective rating of the meaning of different areas of life more accurately. Another major limitation is that no validated questionnaires were used to assess the meaning of the different areas of life. In future studies, standardized questionnaires for measuring values, such as the Valued Living Questionnaire (VLQ) [38] or the Schwartz Portrait Values Questionnaire (PVQ) [39], should be used. Moreover, the generalizability is questionable due to rather small sample size. Another drawback is the missing information on response rates. Unfortunately, we do not know how many patients were invited by their therapists and declined to participate in the online survey. Furthermore, no clear inclusion or exclusion criteria were formulated when recruiting the patients. Comparisons with other countries with other social support systems and countries which were more strongly affected by the COVID-19 pandemic would be interesting.

## 5. Conclusions

Overall, the COVID-19 situation changed the subjective attribution of meaning concerning different aspects of life similarly in therapists as well as patients. While mental and physical health gained subjective importance, the opposite was observed for work.

## Figures and Tables

**Table 1:** Demographic characteristics of the sample.

Characteristics	Patients (*N* = 139)	Therapists (*N* = 222)	Statistics
Gender, *n* (%)			
Female	98 (70.5)	169 (76.1)	X^2^(2) = 2.760; *p* = 0.252
Male	40 (28.8)	53 (23.9)	
Transgender	1 (0.7)	0 (0)	
Age in years, mean (SD)	39.29 (12.18)	50.68 (9.67)	T(1244.6) = 9.828; *p* < 0.001

**Table 2:** Correlation table for the subjective meaning of different areas of life with age at different time points among therapists and patients.

Area of Life	Before COVID-19		During COVID-19		During vs. before COVID-19	
	Therapists	Patients	Therapists	Patients	Therapists	Patients
Work	0.183 **	−0.116	−0.018	−0.146	−0.174 **	−0.057
Relationships	−0.115	−0.123	−0.063	−0.081	0.071	0.063
Acquaintances, friends	0.004	−0.111	0.089	−0.081	0.112	0.029
Leisure time, hobbies	−0.166 *	−0.190 *	−0.031	−0.168 *	0.130	−0.002
Physical health	−0.013	0.194 *	−0.015	0.084	−0.001	−0.170 *
Mental health	−0.003	0.135	−0.063	0.076	−0.075	−0.114

Note: Participants were asked to rate the subjective meaning of the different areas of life from “not important at all” (coded as 1) to “extremely important” (coded as 5). The change in the rating was calculated by subtracting the respective rating before COVID-19 from the rating during COVID-19. Therefore, a positive value means an increase in the subjective meaning of the specific area during COVID-19 as compared to the time before COVID-19. ** The correlation is significant at the level of 0.01 (two-sided). * The correlation is significant at the level of 0.05 (two-sided).
